# Post-abortion care services in Zambian health facilities: a qualitative study of users’ experiences and perceptions

**DOI:** 10.1186/s12905-024-03179-9

**Published:** 2024-07-23

**Authors:** Mwansa Ketty Lubeya, Margarate Nzala Munakampe, Meek Mwila, Musonda Makasa, Moses Mukosha, Choolwe Jacobs, Christabel Chigwe Phiri, Bellington Vwalika, Victor Sichone, Benedictus Mangala, Melissa Mukalumamba Haketa, Andrew Kumwenda, Patrick Kaonga

**Affiliations:** 1 Department of Obstetrics and Gynecology, School of Medicine, University of Zambia, P/B RW1X, Nationalist Road, Lusaka, Zambia; 2 Women and Newborn Hospital, University Teaching Hospitals, Lusaka, Zambia; 3 Young Emerging Scientists Zambia, Lusaka, Zambia; 4 School of Public Health, University of The Witwatersrand, Johannesburg, South Africa; 5 Department of Health Policy and Management, School of Public Health, University of Zambia, Nationalist Road, Lusaka, Zambia; 6 Department of Pharmacy, School of Health Sciences, University of Zambia, Lusaka, Zambia; 7 Department of Epidemiology and Biostatistics, School of Public Health, University of Zambia, Lusaka, Zambia; 8 Levy Mwanawasa University Teaching Hospital, Lusaka, Zambia; 9 Department of Obstetrics and Gynecology, Kitwe Teaching Hospital, Kitwe, Zambia; 10 Zambia Association of Gynecologists and Obstetricians, Lusaka, Zambia; 11 Department of Bioethics, Bloomberg School of Public Health, Johns Hopkins University, Baltimore, MD USA

**Keywords:** Pregnancy, Post-abortion care, Miscarriage, Unsafe abortion, Zambia

## Abstract

**Background:**

Despite attempts to increase Universal Health Coverage, availability, accessibility, acceptability, and quality-related challenges remain barriers to receiving essential services by women who need them. We aimed to explore the experiences and perceptions of women receiving post-abortal care services in Zambia, within a human-rights framework.

**Methods:**

A qualitative case study was conducted between August and September 2021 in Lusaka and Copperbelt provinces of Zambia. Fifteen (15) women seeking post-abortion care services were` interviewed using audio recorders; transcribed data was analyzed using thematic analysis. We report women’s experiences and perceptions of the healthcare system, their experiences of abortion, and healthcare-seeking behaviour. We used the availability, accessibility, acceptability, and quality (AAAQ) framework to understand how women claimed their right to healthcare as they sought and utilized post-abortion care services.

**Results:**

Women who experienced spontaneous abortions delayed seeking health care by viewing symptoms as ‘normal pregnancy symptoms’ and not dangerous. Women also delayed seeking care because they feared the negative attitudes from their communities and the health care providers towards abortion in general, despite it being legal in Zambia. Some services were considered costly, impeding their right to access quality care.

**Conclusions:**

Women delayed seeking care compounded by fear of negative attitudes from the community and healthcare providers. To ensure the provision and utilization of quality all abortion-related healthcare services, there is a need to increase awareness of the availability and legality of safe abortion services, the importance of seeking healthcare early for any abortion-related discomfort, and the provision and availability of free services at all levels of care should be emphasized.

## Plain language summary

Unsafe abortions continue to be an avoidable public health concern both globally and locally. In Zambia, a fifth of maternal deaths are related to unsafe abortions. Unsafe abortions have been defined as any induced termination of pregnancy outside the health facility, performed by untrained health personnel. Several strides have been made including the provision of comprehensive abortion care in all facilities, and the training of health personnel to provide these services at different levels of healthcare. Despite the increased availability of comprehensive abortion care through liberalized abortion laws and regulations and the training of health personnel to provide these services at different levels of healthcare, the need for quality post-abortion care specifically persists. As such, we carried out in-depth interviews to explore the experiences of women who present at healthcare facilities in Lusaka and the Copperbelt provinces in Zambia seeking post-abortion services. It was found that delay in seeking care at a health facility was due to fear that the service is illegal; alongside judgment from health care providers and society for seeking such a service that is generally perceived as evil. The assumption that the cost of receiving such services is high and a general lack of awareness about the service being provided free was also expressed. The study highlighted the need for community awareness of the legal framework on abortions to ensure women access services at any time necessary without fear of being judged, which will consequently reduce the number of abortion-related disabilities and deaths.

## Background

Globally, 10 to 15 per cent of all pregnancies end in miscarriage every year [1, 2]. An average of nearly 73.3 million safe and unsafe (45%) induced abortions occurred each year between 2015 and 2019 [2, 3]. While the majority of miscarriages, stillbirths and induced abortions (97%) occur in low-and middle-income countries, the sub-Saharan region shares this burden disproportionately, with abortion-related mortality estimated at 90 per 100,000 live births [4].

Despite recognizing this need, barriers to quality care continue to deter efforts to attain UHC for women seeking safe abortion and post-abortion care services [5]. Low knowledge of the relatively liberal legal provisions for abortion in Zambia and subsequent unsafe abortions among young girls and women lead to delays in seeking care [6]. Further, the high financial and emotional costs, conservative attitudes towards safe abortion, and hostility or lack of willingness to provide information and services by pharmacy and healthcare workers, in some instances; have all contributed to abortion-related morbidity and mortality [7, 8]. Regardless of whether a woman experienced a spontaneous abortion or safely or unsafely induced abortion, their right to comprehensive abortion care, including post-abortion care remains necessary.

Universal Health Coverage (UHC) goals assert the right to essential and quality healthcare across the life course without enduring financial hardship [9]. This entails ensuring competent health workers who are adequately equipped for the delivery of their work while protecting individuals from the financial consequences of paying for the health services they need. At the 1994 ICPD [1], the declaration that “Every individual had the right to decide freely and responsibly – without discrimination, coercion and violence – the number, spacing and timing of their children, and to have the information and means to do so, and the right to attain the highest standard of sexual and reproductive health” reaffirmed women’s rights to their Sexual and Reproductive Health (SRH). Thus, the right to healthcare cannot be separated from UHC goals and should include the sexual and reproductive health right to access comprehensive abortion care, including post-abortion care.

To firmly realize abortion care-related SRH rights, the government of Zambia has made a long-standing political and policy commitment to address morbidity and mortality associated with abortion; evident from the Standards and Guidelines for reducing unsafe abortion morbidity and mortality in Zambia (2009) [10], and the Standards and Guidelines for Comprehensive Abortion Care in Zambia (2017) [11]. These guidelines are operationalised from the Termination of Pregnancy Act of 1972, which renders Zambia a semi-liberal status-availability of a legal framework allowing TOP under certain conditions, and operationalized guidelines when the legality and availability of abortion are explored.

Although the availability of a legal framework and guidelines that support safe abortion services in Zambia, women continue to face barriers to abortion-care services, including access to safe abortions [10]. Abortion-related stigma on both the provider and the client’s side perpetuates shame, fear, secrecy, guilt, and the subsequent passive care-seeking behaviours. In addition, the Zambian context outrightly disapproves of almost all forms of abortion, citing religious justifications and traditional beliefs [12, 13]. While it is acknowledged that post-abortion care is necessary for both spontaneous and induced abortions, it is necessary to note that women have the SRH right to access this type of care at the health facility without any barriers- including financial hardship. In understanding the experiences of PAC utilization in public health institutions, equity becomes a key consideration [14].

As such, the human rights-based AAAQ Framework illuminates the accessibility, availability, acceptability, and quality of care-related considerations, in trying to understand how women claim or fail to claim their right to health care when seeking PAC services. The human rights-based AAAQ framework was deemed appropriate for comprehensively understanding the health service experiences of women seeking postabortion care, particularly in a context where abortion is severely disapproved. It was anticipated that societal attitudes towards abortion would significantly impact the delivery and utilization of services in this context. By employing a human rights-based framework, we aimed to delve into the intricate dynamics surrounding how women can assert their right to health while seeking postabortion care in the Zambian context. This approach not only allowed for an in-depth exploration of women’s experiences but also facilitated a nuanced understanding of the broader human rights implications inherent in accessing essential healthcare services in such a context. Against this notion, this paper aims to explore the experiences and perceptions of women seeking PAC services in selected health facilities in Zambia.

## Methods

### Study design

A qualitative case study was undertaken to explore the experiences and perceptions of abortion care services among women seeking PAC services. It was conducted in Zambia between August and September 2021 in Lusaka and Copperbelt provinces.

### Study site/setting

Participants were recruited from the University Teaching Hospitals -Women and Newborn Hospital- and Levy Mwanawasa University Teaching Hospitals in Lusaka. These two facilities provide third-level or specialized healthcare in Zambia. In addition, participants were recruited from Kanyama, Chilenje, Chawama, Matero, and Chipata General hospitals, all located in Lusaka city/province. On the Copperbelt province, participants were selected from Kitwe and Ndola Teaching Hospitals (second-level health facilities), adding up to a total of nine (9) health facilities included in the study. These facilities were selected as they are all referral facilities catering to huge populations and they can provide comprehensive abortion care, including PAC services. In all Zambian public health facilities, health services are supposed to be free of any charge. In addition, this study was done before the roll-out of social health insurance in Zambia.

### Sampling and participant recruitment

For this study, women who sought abortion services at the selected health facilities were sampled purposively and based on their willingness to share their abortion experiences and perceptions. They shared experiences of abortion-related physical symptoms, their awareness of the legal framework for abortion and their perceptions of healthcare provisions for PAC services (Table 1).

**Table 1:** Characteristics of women seeking CAC services in selected health facilities

**Participant ID**	**Age Range**^**a**^	**Marital Status**	**No. of children**	**Employment status**	**Number of Pregnancy**	**Induced /spontaneous**	**Onset of abortion**
001	25–29	Married	1	Student Teacher	2^nd^ Pregnancy	Spontaneous	1 month
002	25–29	Engaged/ Unmarried	None	Teacher	1^st^ Pregnancy	Spontaneous	4 months
003	20–24	Married (Polygamous)	3	Unemployed	6^th^ Pregnancy	Spontaneous	3 months
004	n/a	n/a	n/a	n/a	n/a	n/a	n/a
005	40–44	Unmarried	8	Employed	n/a	n/a	n/a
006	25–29	n/a	1	n/a	n/a	Spontaneous	2 months
007	25–29	Unmarried	2	Unemployed	3^rd^ Pregnancy	Induced (cassava branch)	3 months
008	15–19	Unmarried	None	High school 9^th^ grade	n/a	Induced (herbal medicine)	n/a
009	15–19	Unmarried	None	High school 10^th^ grade	1^st^ Pregnancy	Spontaneous	2 months
010	20–24	n/a	None	n/a	1^st^ Pregnancy	Spontaneous	3 months
011	15–19	n/a	None	Student Nurse	1^st^ Pregnancy	Induced (method concealed)	2 months
012	45–49	Married	4	Employed	6^th^ Pregnancy	Induced (pills)	2 months
013	25–29	n/a	n/a	n/a	n/a	Spontaneous	3 months
014	20–24	Married	None	Unemployed	2^nd^ Pregnancy	Spontaneous	n/a
015	20–24	n/a	1	n/a	2^nd^ Pregnancy	Spontaneous	3 months

^a^Age ranges, and not actual age reported to maintain participant anonymity

A total of 15 participants, aged between 17 and 45 years old were included in the study, selected from all women of reproductive age (15–49) accessing post-abortion care in the chosen facilities. Their details are presented in Table 2.

**Table 2:** Characteristics of women seeking CAC services in selected health facilities

		**Characteristic**		**Frequency** **(Out of 15)**		**Percentage** **(Out of 100)**	
**1**		**Marital Status**					
		Married		4		27	
		Unmarried		5		33	
		n/a		6		40	
**2**		**Age**					
		Below 18		1		7	
		18–24		6		40	
		25–34		5		33	
		35–49		2		13	
		n/a		1		7	
**3**		**Employment status**					
		Unemployed		3		30	
		High school		2		13	
		Employed		4		27	
		College/University student		2		12	
		n/a		5		33	
**4**		**Number of children**					
		No children		6		40	
		Children		7		47	
		n/a		2		13	
						0	
**5**		**Abortion**					
		Induced		4		27	
		Spontaneous		9		60	
		n/a		2		13	
**6**		**Onset of abortion**					
		1^st^ month		1		7	
		2^nd^ month		2		13	
		3^rd^ month		5		33	
		4^th^ month		1		7	
		n/a		4		27	
**7**		**Number of Pregnancy**					
		1^st^		4		27	
		2^nd^		2		13	
		3^rd^		1		7	
		6^th^		3		20	
				5		33	
**8**		**Facility**					
		Facility 1- Lusaka		3		20	
		Facility 2- Lusaka		2		13	
		Facility 3- Lusaka		1		7	
		Facility 4- Lusaka		2		13	
		Facility 5		2		13	
		Facility 6- Copperbelt		1		7	
		Facility 7- Copperbelt		2		13	
		Facility 8- Copperbelt		1		7	
		Facility 9- Copperbelt		1		7	

### Data collection

Trained research assistants composed of medical students, nurses and medical doctors conducted the interviews. Interviews were conducted in English, Nyanja or Bemba. Research assistants were briefed on ethical research practices, including cultural appropriateness while interacting with women identified within the departments providing post-abortion care services, especially considering how sensitive this topic is. Interviews were steered with interview guides and were recorded using digital recorders after permission was sought from the participants. Questions on the events that led to seeking care, what was done on arrival at the facility, the services received, and the women’s perceptions of the legal status of abortion in Zambia were asked. The investigators of the study stored recorded interviews.

### Data management and analysis

Audio-recorded interviews were transcribed (verbatim) and translated, and transcripts were read alongside any notes taken during fieldwork. The study investigators listened to selected recordings while reading the translated transcripts to ensure that meanings were not lost, and to ensure consistency. All the data was analyzed manually using thematic analysis, which was deemed appropriate for analyzing and reporting patterns within the data collected. Initially, open coding was done by three of the authors (MKL, MNM and MM) separately, and an initial code list was developed. In developing a single code list, discussions ensued to assess any variations in coding and any other additional codes. After that, coding of the rest of the transcripts was done, with the allowance to add any emergent themes based on the additional coding. Informal discussions and fieldwork experiences were also drawn upon in the analysis and presentation of results. The Human Rights-based Availability, Accessibility, Acceptability and Quality (AAAQ) criterion is drawn upon to interpret and understand how women claim their right to health care as they seek and utilize post-abortion care services.

## Results

We present women’s experiences of abortion and perceptions about post-abortion care service utilization in selected health facilities in Zambia. We grouped the experiences and perceptions into three major themes- (i) women’s experiences of abortion and their healthcare-seeking behaviour, (ii) perceptions of the healthcare system and their knowledge of the legality of abortion, and lastly, (iii) their views on possible measures to improve post-abortion care services.

### Experiences of abortion- signs and symptoms

Women who had reported a spontaneous abortion mentioned noticing physical signs and symptoms such as abdominal pains, bleeding with clots, vomiting, loss of appetite, and headache. Initially, some women did not realize that they were going through an abortion, despite having these symptoms as they assumed it was a normal occurrence in pregnancy. One participant, when asked to describe how she knew that she had an abortion, said:

“I started having strong abdominal pains around 03:00 am, somewhere there, I thought it was normal pain when you are undergoing pregnancy, issues change here and there,” (Participant 2, 25-29yrs, unmarried, teacher, spontaneous).

While pregnant women were encouraged to notice any danger signs during pregnancy, one woman mentioned being worried about the status of her pregnancy after noticing heavy bleeding. She said:

“(...) when I was bleeding on Tuesday though, I went to the market in the evening to get a pregnancy test kit to check if I was still pregnant. The kit recorded two lines showing I was still pregnant, so I thought the pregnancy was just fine. ” (Participant 14, 20-24yrs, married, unemployed, spontaneous).

Respondents who had no prior experience with any abortion-related event had an initial shock reaction when they saw what they were going through, especially when it persisted. The hesitancy to take these signs seriously or wait to see if signs such as heavy bleeding would persist, caused a delay in seeking medical care. Women reported that the symptoms had started much earlier, mostly at night. Self-medication such as analgesics to manage the pain accentuated the delay. Responses from women on whether they had experienced a miscarriage before or not, varied; however, generally, they only resorted to visiting the facility as their symptoms worsened, or in an emergency state as narrated by a participant:

“I just ignored it, but the pain continued until around 06:00 am. I decided to come to the hospital, and the baby was almost out.” (Participant 2, 25-29yrs, unmarried, teacher, spontaneous).

### Events leading to the onset of the spontaneous or induced abortion, and sources of abortifacients

All women attributed the onset of abortion to several factors. Women who said they took or did something that induced the abortion knew the “cause”, although the willingness to reveal this information was low. They narrated hearing of individuals pricking the uterus with a “cassava” branch or drinking herbal or unknown concoctions to induce the termination of a pregnancy. One 45–49-year-old woman with four children mentioned taking pills that she bought from a pharmacy. When asked what she took to terminate the pregnancy, she said: “*I don't know what medicine they wrote for me, but there were only four tablet*s” (Participant 12, 45-49yrs, married, employed, induced), citing how she bought the pills after receiving a prescription from a doctor at one of the health facilities.

Women also reported their feelings towards their pregnancy, citing how these feelings often led to the decision to induce the abortion or affected their response to danger signs when experiencing a spontaneous abortion. A participant narrated how she was not ready to keep the pregnancy after finding out about her partner’s infidelity. Reasons for termination and the method used varied among the different participants. A safe termination was an option, but she sought unsafe options to terminate her pregnancy. It is worth noting that these methods were readily available in the community, from various sources such as family members, traditional healers and pharmacies. When asked what methods she used to terminate the pregnancy, the participant had this to say:

“I removed [terminated] the pregnancy. I used a stick which looked like a cassava tree branch; the person who impregnated me wasn’t telling me the truth, and he has a wife. ” (Participant 7, 25-29yrs, unmarried, unemployed, induced)

Similarly, a 15–19-year-old grade 9 pupil narrated her story, saying: “*Am here because of abdominal pain, I drank herbal medicine, [which] my friends’ grandmother made for me*” (Participant 8, 15-19yrs, 9^th^ grade, induced). While some participants can mention that they took concoctions to induce the abortion, the details of what they took exactly were not forthcoming. Despite women resorting to clandestine and unsanctioned sources of abortifacients, safe abortion services are available in some local health facilities. A participant who had a safe abortion said:

“One of the gentlemen I work with (…) is the one who explained to me to say I should go to the hospital, they now offer the service” (Participant 8, 15-19yrs, 9^th^ grade, induced)

Women who experienced the sudden onset of an abortion (spontaneous abortion) noted various “potential causes”, or at least factors thought to be contributors to the spontaneous abortion occurrence. Such causes included stress, experiencing problems, general sickness, and performing household chores or duties such as cleaning the house, laundry and cooking. Without completely knowing what might have caused the abortion, women recalled the last activity they engaged in before the onset of the abortion. A young woman who narrated what she thought triggered her initial discomfort said:

“I don’t know what caused it... because yesterday as I was washing, I had taken some flying fish [alcoholic beverage-beer] that my friend brought for me. After I drank and my friend left, I started to feel my stomach hurting [stomach pain] (...) yes, it was just yesterday, and last of last week [two weeks ago] (...) I took two bottles of Savanna [alcoholic beverage-cider] (Participant 3, 20-24yrs, married, unemployed, spontaneous)

### Perceptions of post-abortion care and knowledge of the legality of abortion

The women using post-abortion care services – whether they had a spontaneous or induced abortion highlight the critical role played by the health care system in mitigating complications related to abortions. They also narrated their experiences of service utilization.

#### Availability of supplies and the cost of services

Hospital supplies enhance the provision of quality healthcare services to clients. However, women mentioned that supplies were not readily available when they needed them. Consequently, the women reported out-of-pocket expenditures to receive post-abortion care services, when all services in public health facilities in Zambia are supposed to be free of charge. Some clients reported that health facility staff asked them to pay for services such as obstetrics ultrasound, laboratory investigations, drugs, and other consumables such as cotton wool, gloves, disinfectants, and other infection-prevention materials. Women recalled spending this money at the expense of vital needs such as food. When asked about the costs incurred while accessing abortion-care services, a 25–29-year-old married woman had this to say:

“….. I’m just disappointed that I have to umm pay for services which are not supposed to be paid for and again I was not given any antibiotics, I have to buy myself……. It was going to be better if maybe I just stayed home. I go to a chemist I explain there to the physician, and then they just prescribe...other people just opt to stay away. They just decide not to go to hospitals because they know that even if they go there [hospital] there isn’t any medication they will be given. (Participant 1, 25-29yrs, unmarried, student teacher, spontaneous).

Another woman, who could not access post-abortion care until she had enough money to buy supplies so that the clinic could use them for her procedure, had this to say:

“.....they [HCW] told me to call people from home to buy for me what is needed to be used because there wasn’t anything to use hence they couldn’t do anything without it [consumables].......they told me to buy *jik* [bleach], gloves, plastic and cotton" (Participant 6, 25-29yrs, 1 child, spontaneous).

In some instances, women sought financial assistance from family or friends to cover their costs. The women from whom healthcare providers sought payment for services and supplies noted how these payment requests delayed service access and utilization. When asked how easy it was for her to find the money, one woman said: “*Yes [it was difficult], it is what they are currently discussing [how to provide the payment]…they said we should go home and organize [find money to pay for services]”* (Participant 1, 25-29yrs, unmarried, student teacher, spontaneous). Particularly in consideration of socioeconomic hardships left in the wake of the COVID-19 pandemic, payments for services were noted as even more challenging for women. They said:

“...umm with this COVID-19 pandemic which is hitting us, money, of course, is the talk of the day that it is not easy to find. I had to borrow from someone for me to get that money to find myself here.” (Participant 1, 25-29yrs, unmarried, student teacher, spontaneous).

“So from yesterday since I arrived [at the health facility], coz my phone was off until now, that is when I asked someone to put my sim card in their phone, so they [relatives] sent the money.” (Participant 6, 25-29yrs, 1 child, spontaneous).

In some cases, clients reported making payments for services directly to the provider, and these payments were for the service and not necessarily direct costs for tests or consumables. A woman who made a direct payment to a service provider lamented this decision. She said:

“He (HCW) requested that I give him a K30 (USD 1.5) for a test they don't have at this facility…...for him to help me. So I was wondering if someone in this facility has the kind of treatment they can only give out to patients when you give them something.” (Participant 1, 25-29yrs, unmarried, student teacher, spontaneous).

#### Quality of services received at the health facility

Women receiving post-abortion care cited mixed feelings regarding the quality of services they received at the health facilities. Some clients described satisfaction with the services provided, despite expecting the services to be of poor quality. They expressed shock as they narrated their experience of good service provision, which exceeded their pre-judgements. When asked about the care she received, a woman had this to say:

“It is amazing, it’s nice I can’t complain (...) maybe I was the lucky one, people complain, no doctor they don’t do that and that. But for me, it was okay; everything was done on time, I’m even fit, and I can move.” (Participant 2, 25-29yrs, unmarried, teacher, spontaneous).

“I was attended to properly when I arrived; they came fast enough, I was checked, they removed some clots that remained….at the time there was no power...so they switched on the generator, and my womb was cleaned.” (Participant 15, 20-24yrs, 2^nd^ pregnancy, spontaneous).

However, for other women, the experience of the care received was not very satisfactory as this affected their perception of post-abortion care in Zambian health facilities.

“Yeah, so I think umm because you need that thing [post-abortion care], you will sometimes find it difficult to like negotiate because that person will just go like ‘maybe you just give me K500 (USD 30) then I will help you out. Then you who[ever] is in need absolutely have [has] no choice in trying to negotiate with that person, but just to find that money and give that person [HCW] so that you get the services that you need” (Participant 1, 25-29yrs, unmarried, student teacher, spontaneous).

#### Knowledge of the legality of abortion at the individual and community level

Disbelief and misinformation surrounded the legal status of abortion, with some of the participants mentioning that they were aware of the law before seeking services or at the facility while receiving post-abortion care services. The lack of knowledge on the legality of seeking abortion services under certain conditions, led to disbelief when it was mentioned during the discussions. Informants indicated that they were not very familiar with the legal framework but were sure of the possibility of incarceration associated with terminating a pregnancy. Examples of incarcerated individuals who performed unsafe abortions in the community were cited as evidence of the criminality of seeking and performing abortions.

At the community level, abortion was completely frowned upon and was not linked to the legal status but to whether it was morally acceptable by the community or not. This deterred women from seeking safe abortion services and left them with unsafe options such as ingesting homemade or herbal concoctions or purchasing over-the-counter medication without a prescription to induce the abortion. When asked about the legal status of abortion services in Zambia and whether they were permissible in the community where she lives, a woman said:

“ (…) they say that it is not allowed (…) in our African setting, it is not allowed so most of the times, people just perform them on their own in the house (…) they usually go to the elders of the community that are knowledgeable about the drugs they use.” (Participant 12, 45-49, married, employed, induced).

Ironically, some informants affirmed their knowledge of the legal status of abortion in Zambia. However, being knowledgeable did not provide sufficient grounds to seek a safe abortion service, as it was morally unacceptable by community standards. While stating that some people were aware of the law, it was also noted that low utilization of safe abortion services was surrounded by ignorance of the legal status of the abortion law (Termination of Pregnancy Act of 1972). Thus, women were left with very few options and usually opted to secure alternatives from trusted members of the community. A woman who was asked about the legal status of abortion in Zambia and what people in the community thought about the termination of pregnancies said:

“Of course, I know about abortion; it is now legalized. If you don’t like keeping the baby, you go to the doc [medical doctor], you take whatever [inducements/abortifacients] (…) I have never done that [but] that’s what I have heard when you want to abort, you go to the doctor, you go to the nearest hospital, you agree [consent], you sign somewhere (…) they allow. (…) Now out of ignorance, since I am a teacher by profession, these are the things we meet [deal with]. We come across…, especially for pupils; they just seek these women…the elderly in the community. They [the elderly] give them medicine, whatever the case and everything is done just there and then, or they just go straight to the pharmacies, and they buy (…).” (Participant 2, 25-29yrs, unmarried, teacher, spontaneous).

### Suggestions from women on how to reduce unsafe abortions and increase the utilization of safe abortion services

The discussants were also asked to describe what they thought would help to reduce the cases of unsafe abortion in their communities. Most views reflected the need to increase sensitization efforts, thus increasing awareness of the legal status of abortion in Zambia. The benefits of increasing awareness of the legal status of abortion were noted as key in the prevention of unsafe abortions, provided the availability of services. While it was reported that ignorance prevented women, especially adolescents and young women, from seeking safe abortion services, knowledge alone was not sufficient, as there was also a need to decriminalize abortion at the community level. A young, woman who had induced her abortion said;

“I am not sure, I just hear them talk, we just see [hear] that if you have aborted (…) when they see you, they arrest you (...) if you have aborted, the police come to arrest you” as she narrated her perception of how the community views the termination of a pregnancy” (Participant 7, 25-29yrs, unmarried, unemployed, induced).

Another young woman said:

“it’s illegal. They don’t allow it because it’s the same as killing a person (...) they know that they don’t allow [terminating a pregnancy] in Zambia because so many things happen to you, and it’s just like killing another person.” (Participant 9, 15-19yrs, unmarried, high school, spontaneous).

Historically, increasing awareness of such services as those related to sexual and reproductive health to young people has been contentious. It was seen to promote risky and promiscuous behaviour among the youth and the criminality associated with the act. However, a double-burrowed approach that targets both young people and other members of their communities was noted as necessary to reduce the stigma associated with seeking abortion services. A participant who was asked what needs to be considered to improve the utilization of safe abortion services said:

“I think they [potential users] need to be educated (…) [through], especially schools. You know, everything starts from school, when you educate people in schools; they take the information to the community. So I think, unless otherwise, they [community members] will say you are promoting these little ones [promoting risky or promiscuous behaviour], but you can even meet people from the same communities in the markets (…)” (Participant 2, 25-29yrs, unmarried, student teacher, spontaneous).

After reporting that they were charged for various health services during their visit to the health facility, women noted that paying must be stopped. Prohibition of such payments would ensure that more women were more capable of accessing post-abortion care services and would ensure overall satisfaction with the quality of services rendered. A young woman said:

Interviewer: How do you think the hospital can improve its service provision for people who come here seeking abortion services?

Participant 8: They should stop making people pay the K200s [*about USD 11 at the time of data collection*] (15-19-yrs, unmarried, unemployed, no children, induced).

## Discussion

The experiences and perceptions of women seeking post-abortion care services were centred on women’s experiences of abortion and their healthcare-seeking behaviour, the ways they navigated the healthcare system once they decided to seek post-abortion care services, their views on the legal status and acceptability of induced abortion and how post-abortion care services can be improved. Keeping these findings in mind, the human rights-based AAAQ criterion is drawn upon, to understand how and if women claim their right to health care as they seek and utilize post-abortion care services in Zambia [15]. Using the right to health as the central standard for assessing health care imposes four essential standards on health services, availability, accessibility, acceptability, and quality of health care services.

The AAAQ standard reaffirms that the “a*vailability of services requires that public health and healthcare facilities are available in sufficient quantity, taking into account a country’s developmental and economic condition*” [15]. In Zambia, women who presented to the health facility for post-abortal care acknowledged the availability of services in district-level health facilities, even though some had been referred from primary care facilities. Campbell et al. [16] used the signal function to analyze abortion care services, to illustrate its use in Zambia. They found that under a minimal scenario without considering family planning, 14.8% of Zambian facilities met the basic criteria for basic post-abortion care and 2.7% for post-abortion care-modified abortion care functions.

However, the reports of low availability of abortion-related services are not unique to Zambia but are characteristic of many other LMICs. It was observed that the availability of human and material resources are significant factors in improving healthcare services while providing individuals with the reassurance of readily available materials when they present to seek care. Regarding the legality of safe abortion services, women showed little or no knowledge of the Termination of Pregnancy Act of 1972, despite Zambia being one of the few countries with the most liberal abortion laws in the sub-Saharan region. The legality of abortion at the community level was also marred by a lack of information about the semi-liberal law. Geary et al. [17] reported this too, suggesting that increasing awareness about the legal status of abortion would lead to more favourable attitudes about abortion services in general [5, 18].

For the accessibility construct, four overlapping domains are unpacked: non-discrimination, physical, economic and information accessibility [15]. Our study comprised facilities in urban areas in Lusaka and the Copperbelt provinces of Zambia, which have both urban and peri-urban populations they cater to. Women, who predominantly came from urban and peri-urban areas, cited some challenges with accessing services for post-abortion care. This assertion slightly differs from what Campbell et al. [16] found using population-level data for Zambia, as they noted how access to abortion care services for urban dwellers was much better than access in the more rural areas. These conclusions were based on survey data, and thus we argue that collecting data on personal experiences enabled us to gain a deeper understanding of what women go through, beyond the numbers that are usually collected in surveys and censuses.

Further, despite living in urban areas, some of these women may consist of the population living in high-density slums of these urban areas. In this study, women interviewed were vulnerable to facing additional consequences such as being charged for services and care from providers, especially if they were not ready to pay for the services they needed. Some of the women lamented the lack of resources needed to access healthcare, highlighting this as a significant impediment to health-seeking. The failure to pay for services on their own prompts women to seek alternative sources of help, which unfortunately jeopardizes their health. Leone et al. [6] found that some women seeking abortion care were vulnerable to debt and poverty when faced with unexpected costs for multiple referrals and follow-up visits during abortion-related care [14]. This partially explains the late presentation to a point where the situation is dire, leaving almost no option but to visit the health facility. In addition, women’s lack of information about their legal right to seek safe abortion as well as post-abortion care services must be emphasized, to enable women to claim their right to healthcare. The inability to pay for services in some cases is also indicative of the importance of socioeconomic empowerment to increase access to essential services for women [19], especially adolescents and young girls [20].

The acceptability construct “*requires that health services are ethically and culturally appropriate, i.e. respectful of individuals, minorities, peoples, and communities, and sensitive to gender and life-cycle requirements*”[15]. Acceptability of post-abortal care services in the Zambian setting was still low, particularly in instances where women had tried something to induce an abortion before going to the health facility [18], evident also by women who reported not knowing abortion was legal, but sought post abortion care. Abortion, in general, is surrounded by religious and traditional views that are suspicious of both induced and spontaneous abortions, considering the sin and shame associated with abortion in a constitutionally declared Christian nation [13, 20]. The need to be discrete was consistent with a study in South Africa where women shunned care due to fear of their abortion being discovered by others, and avoiding labels such as “killers, sinners, and mothers of devils” [21]. In addition to concerns about people within their community finding out about the induced abortion and facing possible incarceration was also noted as a key barrier to seeking care in time. The delay in seeking health care after experiencing discomfort may also be culturally motivated, where women are supposed to be “strong” and endure minor discomforts during pregnancy, as others have noted [22]. Whether cultural or religious, such norms must be deconstructed, to increase awareness of seeking care for any discomfort during pregnancy. In addition, women must also be reminded of their capacity to seek legal and safe abortion services, instead of opting to visit the health facility for the management of complications. Reduced acceptability of care-seeking led many to seek alternative care before resorting to the health care system to manage advanced symptoms.

We also delved into issues surrounding the quality of care in accessing abortion care services. Quality “*requires that health services must be scientifically and medically appropriate and of the highest quality*” [15]. Perpetual stock-out of consumables, such that clients had to spend out of pocket to supply consumables needed to receive the service, was a recurring experience for women seeking care services. While this particular occurrence reduces access to services, women viewed this as significantly compromising the quality of services provided, as it discriminates against clients who may not have money when presenting to the health facility. We note reports of women receiving care up to 24 hrs after presenting to the health facility or being sent back home after a failure to produce funds for consumables as a precursor to being attended to. Even though this study did set out to specifically measure signal functions to assess the quality of post-abortion care, an earlier assessment in Zambia [17] shows that the quality of care still falls short. These findings are consistent with experiences in other countries in the region like Zimbabwe, where the quality of care is below the recommended threshold [23], thus continuous quality improvement efforts across the healthcare system are necessary.

### Strengths and Limitations

Although experiences and perceptions were captured from women seeking post-abortion care services in selected facilities, a smaller number than anticipated (due to refusal by other users of PAC services to take part in the study) reduces transferability [24]. This was attributable to several factors; the sensitive nature of the topic under discussion and the discomfort experienced by women as they shared information related to their abortion experiences because abortions are highly criminalized in the communities. Therefore, some women who could have used unsafe methods to terminate their pregnancies might have reported that they lost the pregnancy spontaneously due to social desirability. We also covered sites that report the highest rates of abortions in the country, which may pull transferability towards high-rated abortion-care-seeking contexts.

This study reviewed women’s experiences as they access PAC services, a sensitive topic. Despite this, participants varied by age, term of pregnancy and whether the abortion was spontaneous or induced, enriching the representation of the experiences and perceptions captured, and increasing credibility. A description of the study context was done to an extent, which allowed the research team to improve the transferability of the findings. In addition, standard procedures were used to collect and analyze the data, increasing dependability [25]. Confirmability was increased during the analysis, as three researchers were involved in the analysis and sense-making process, and the overall trustworthiness of the study findings. Some interviews were brief because some women got very emotional during the discussions and could not continue with the study. In addition, findings are limited to those women who made it to the facility for post-abortion care. It is therefore likely that many other women were dealing with abortion-related morbidity (or even mortality) who did not seek care at the facility. However, we brought out some key issues that may delay care on both client and service provider sides from the women’s perspective and were consistent with what others have found.

## Conclusion

We found that women seeking post-abortion care services experienced barriers to accessing quality health services from the individual level to the community, and facility levels as well. These findings entail that there is still a need to continue strengthening sexual reproductive health services, specifically post-abortion care to ensure that women easily access these services. There is also an urgent need to actively fight community and facility-based stigma around seeking abortion care services so that women can opt for safe abortion care initially, to avoid unsafe procedures and then subsequent PAC. Further, there should be continued community education around Zambia’s TOP act in a culturally sensitive manner to improve the uptake of general abortion care and specifically PAC services, to aid the attainment of SGD # 3 by 2030.

## Data Availability

The raw data generated and/or analyzed during the current study are not publicly available but are available from the corresponding author on reasonable request.

## Abbreviations

AAAQ: Availability, Accessibility, Acceptability and Quality
CAC: Comprehensive Abortion Care
LMIC: Low-middle-income countries
PAC: Post-Abortion Care
SDG: Sustainable Development Goal
SRH: Sexual and Reproductive Health
TOP: Termination of Pregnancy
UHC: Universal Health Coverage
